# P-246. *Candida auris* Central Line Associated Blood Stream Infection Trends in Chicago Acute Care Hospitals, 2018-2023

**DOI:** 10.1093/ofid/ofae631.450

**Published:** 2025-01-29

**Authors:** Vaishali Chundi, Kelly Walblay, Shane Zelencik, Do Young Kim, Stephanie R Black

**Affiliations:** Chicago Department of Public Health, Chicago, Illinois; Chicago Department of Public Health, Chicago, Illinois; Chicago Department of Public Health, Chicago, Illinois; Chicago Department of Public Health, Chicago, Illinois; Chicago Department of Public Health, Chicago, Illinois

## Abstract

**Background:**

The emergence of antifungal resistance and propensity of *Candida auris* to cause healthcare facility outbreaks and severe outcomes in patients has deemed it a target pathogen for public health mitigation efforts. Data sharing between the Chicago Department of Public Health (CDPH) and the National Healthcare Safety Network (NHSN) allows for the surveillance of central line associated blood stream infections (CLABSIs) in Chicago healthcare facilities. We reviewed the *Candida* species associated with CLABSIs to assess *C. auris* contribution to CLABSIs in Chicago over time.

**Figure:**
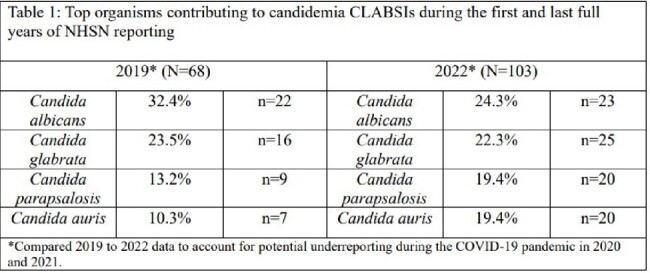

**Methods:**

Data was reported by 32 acute care hospitals (ACHs), including five long- term ACHs between July 1, 2018 and June 30, 2023. All but one ACH provided data for the entire reporting period. We examined trends of central line associated candidemia over time, specifically *C. auris*. A Fischer's exact test was calculated to determine if the proportion of *C. auris* significantly differed from 2019 to 2022. Patient demographics and outcomes were summarized for individuals with *Candida* species grown on culture.

**Results:**

From July 1, 2018-June 30, 2023, 2,362 CLABSIs were reported of which 460 (19.5%) were associated with *Candida* species. The median age of patients with *Candida* CLABSIs was 60 years (IQR: 45-69 years), and over half (58.7%) of individuals were male. Among 160 patients who had a *Candida* associated CLABSI and cause of death data available, candidemia contributed to 18.8% of deaths. The contribution of *C. auris* to candidemia CLABSIs increased from 10.3% in 2019 to 19.4% in 2022 (p=.1353) and *C. parapsilosis* increased from 13.2% to 19.4%, while *C. albicans* declined from 32.4% to 24.3%, respectively (Table 1).

**Conclusion:**

*Candida* species make up close to 20% of CLABSIs reported by Chicago ACHs during 2018-2023, with *C. auris* contributing to nearly one-fifth of them in 2022. While the growing proportion of CLABSIs associated with *C. auris* from 2019 to 2022 is not statistically significant, it has clinical significance, and demonstrates that healthcare facilities should emphasize critical assessment of need for central line placement and infection control interventions, such as hand hygiene and environmental cleaning, to prevent further spread of *C. auris*.

**Disclosures:**

**All Authors**: No reported disclosures

